# Tetracycline response element driven Cre causes ectopic recombinase activity independent of transactivator element

**DOI:** 10.1016/j.molmet.2022.101501

**Published:** 2022-04-19

**Authors:** Kenneth T. Lewis, Lily R. Oles, Ormond A. MacDougald

**Affiliations:** 1 University of Michigan Medical School, Department of Molecular & Integrative Physiology, Ann Arbor, MI, USA; 2 University of Michigan Medical School, Department of Internal Medicine, Ann Arbor, MI, USA

**Keywords:** Ectopic Cre activity, Tetracycline response element, pTet, TRE-Cre, tetO7-Cre, AdipoChaser

## Abstract

**Objective:**

Tamoxifen is widely used for inducible Cre-LoxP systems but has several undesirable side effects for researchers investigating metabolism or energy balance, including weight loss, lipoatrophy, and drug incorporation into lipid stores. For this reason, we sought to determine whether a doxycycline-inducible system would be more advantageous for adipocyte-specific Cre mouse models, but serendipitously discovered widespread ectopic tetracycline response element Cre (TRE-Cre) recombinase activity.

**Methods:**

Adipocyte-specific tamoxifen- and doxycycline-inducible Cre mice were crossed to fluorescent Cre reporter mice and visualized by confocal microscopy to assess efficiency and background activity. TRE-Cre mice were crossed to stop-floxed diphtheria toxin mice to selectively ablate cells with background Cre activity.

**Results:**

Tamoxifen- and doxycycline-inducible systems performed similarly in adipose tissues, but ectopic Cre recombination was evident in numerous other cell types of the latter, most notably neurons. The source of ectopic Cre activity was isolated to the TRE-Cre transgene, driven by the pTet (tetO7) tetracycline-inducible promoter. Ablation of cells with ectopic recombination in mice led to stunted growth, diminished survival, and reduced brain mass.

**Conclusions:**

These results indicate that tamoxifen- and doxycycline-inducible adipocyte-specific Cre mouse models are similarly efficient, but the TRE-Cre component of the latter is inherently leaky. TRE-Cre background activity is especially pronounced in the brain and peripheral nerve fibers, and selective ablation of these cells impairs mouse development and survival. Caution should be taken when pairing TRE-Cre with floxed alleles that have defined roles in neural function, and additional controls should be included when using this model system.

## Highlights

• TRE-Cre causes ectopic recombinase activity, particularly in neurons.
• TRE-Cre leak is independent of rtTA or doxycycline treatment.
• Ablating cells targeted by ectopic TRE-Cre recombination diminishes mouse survival.
• Validating extent of TRE-Cre leak with a given floxed allele is an important control.

## Graphical abstract

**Proposed control procedure for doxycycline-inducible mouse models.** Diagrammatic representation of a two-step approach to control for undesirable off-target effects in doxycycline-inducible TRE-Cre mice. First, tissues of TRE-Cre^−^^/−^/rtTA^+/^^−^/allele^fl/fl^/(+)Dox and TRE-Cre^+/^^−^/rtTA^−^^/^^−^/allele^fl/fl^/(+)Dox mice should be compared to TRE-Cre^−/−^/rtTA^−^^/^^−^/allele^fl/fl^/(+)Dox controls and screened for ectopic Cre recombination or transactivator off-target effects. If mice with the floxed allele of interest pass screening, experimental TRE-Cre^+/^^−^/rtTA^+/^^−^/allele^fl/fl^/(+)Dox mice should be controlled with TRE-Cre^+/^^−^/rtTA^−^^/^^−^/allele^fl/fl^/(+)Dox mice.

**Figure:**
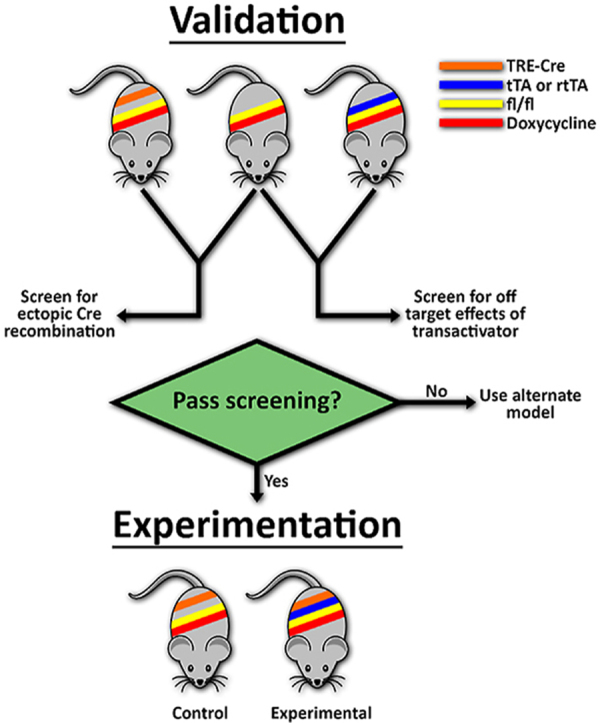

## 1 Introduction

Cre-LoxP technology has revolutionized our ability to determine tissue and cell-type specificity of numerous molecular pathways by allowing for conditional induction or deletion of genes. The Cre-LoxP system requires two components; Cre recombinase driven by a cell-type or tissue specific promoter, and a LoxP-flanked (floxed) allele of interest. The past three decades have seen the development of a vast library of mouse Cre-driver strains and floxed alleles to enable near limitless combinations of conditional gene expression systems. In order to lend temporal control to conditional mutation models, inducible Cre-LoxP systems were developed which use small molecule ligands to regulate Cre expression.

**Figure 1:**
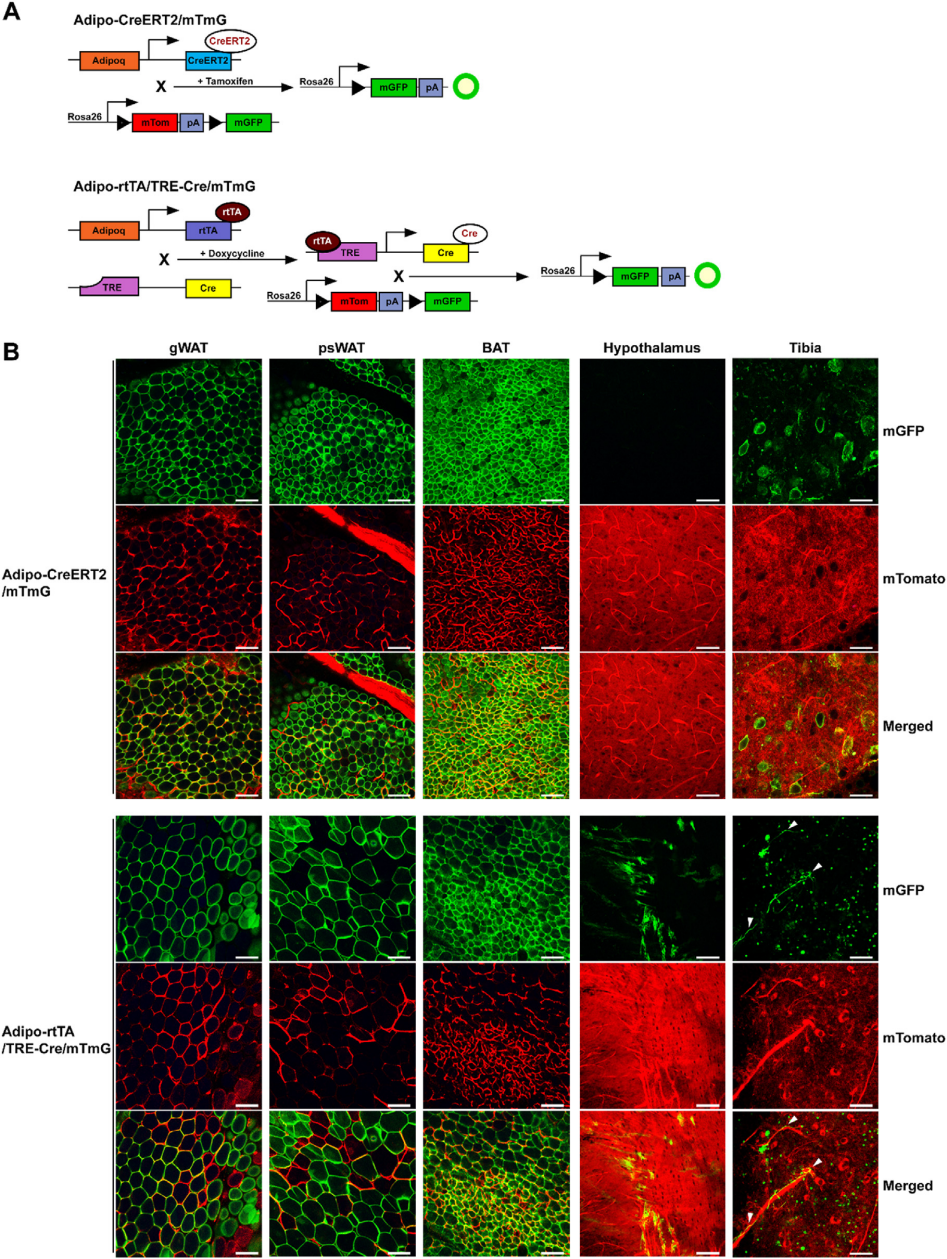
**Doxycycline-inducible Adipo-rtTA/TRE-Cre recombination has comparable efficiency to tamoxifen-inducible Adipo-CreERT2, but with additional off-target recombination.** Diagrammatic representation of Adipo-CreERT2/mTmG (Top) and Adipo-rtTA/TRE-Cre/mTmG (Bottom) inducible adipocyte-specific mouse models with genes represented as rectangles and proteins as ellipses (A). Representative confocal micrographs of fresh whole-mount tissue showing Cre recombination efficiency in gonadal white (gWAT), posterior subcutaneous white (psWAT), and interscapular brown adipose (BAT) depots, and ectopic Cre recombination in brain (hypothalamus) of female Adipo-CreERT2/mTmG (N = 3) vs Adipo-rtTA/TRE-Cre/mTmG (N = 3) mice (B). mGFP is represented in green, mTomato is represented in red, and white arrowheads point to nerve fibers. Scale bar = 100 μm, age 22–47 wks.

The most widely used inducible Cre system features a chimeric Cre recombinase fused to a mutant estrogen receptor (CreERT), which is activated by metabolites of the selective estrogen receptor antagonist Tamoxifen, but not by physiological concentrations of estrogen [[1], [2], [3]]. Like constitutive Cre-LoxP systems, CreERT is driven by a cell-type or tissue specific promoter and exerts its control by excising floxed DNA. Inactive CreERT forms a heterodimer with heat shock protein 90 (HSP90) and is sequestered to the cytoplasm. Only once the tamoxifen metabolite 4-OH-tamoifen binds to CreERT, can the protein translocate to the nucleus and bind LoxP sites and catalyze recombination (Figure 1A) [[4], [5], [6]]. While CreERT and its more active derivative CreERT2 are widely used and allow tight spatiotemporal gene regulation in tissues, the biological activity of tamoxifen itself raises some concerns for its use in experimental animals. Tamoxifen administration causes marked weight loss and lipoatrophy regardless of administration route and has a significant mortality rate at commonly used doses [7,8]. While the side effects of tamoxifen can be reduced by limiting the amount administered and/or duration of treatment, this may also lower frequency of Cre recombination and complicate interpretation of experimental results. Furthermore, since tamoxifen is a steroid derivative, it readily dissolves in lipid. In dosed animals, adipose acts as a reservoir for tamoxifen, which can be released at later timepoints in sufficient quantity to induce CreERT-mediated recombination [7].

Alternative inducible Cre-LoxP systems that use tetracycline-family antibiotics to repress or induce Cre expression are known as Tet-off and Tet-on, respectively. These systems incorporate three components; a tetracycline-responsive element (TRE; pTet; tetO7) that drives Cre expression (TRE-Cre), a cell-type or tissue specific transactivator that binds TRE to either repress or induce transcription (tTA and rtTA, respectively), and a floxed allele (Figure 1A) [9]. For Cre induction in the Tet-on system, a tetracycline-family antibiotic, typically doxycycline, binds the reverse transactivator (rtTA), which causes it to bind TRE to drive Cre expression. The opposite is true in the Tet-off system, in which tTA constitutively binds and activates TRE until doxycycline causes its dissociation, leading to cessation of Cre expression. Unlike tamoxifen, doxycycline readily forms an aqueous solution and can be simply administered to the animal in their drinking water or food. Additionally, doxycycline offers tighter temporal control than tamoxifen. Whereas overnight washout is possible with doxycycline, some level of CreERT2 nuclear translocation has been observed following two months washout, likely due to sequestration of tamoxifen within lipid stores [7,10]. Furthermore, doxycycline administration in food is not accompanied by weight loss and high mortality rates as is seen with tamoxifen [11]. These properties make doxycycline-inducible systems ideal for studies involving metabolism, body composition, or adipose tissues. However, doxycycline-inducible Cre-LoxP models are not without their drawbacks. In culture, doxycycline exerts a cytotoxic effect at doses recommended for the Tet-on system [12]. In mice, doxycycline doses commonly used to induce Cre recombination are sufficient for antibiotic activity, which can induce dysbiosis and disrupt mitochondrial protein production [[13], [14], [15]]. However, these disadvantages can be readily controlled for exposing both experimental and control animals to the same doxycycline treatment.

In this study, we sought to compare tamoxifen and doxycycline inducible Cre-LoxP systems for use in adipose tissue research, but unexpectedly observed ectopic Cre recombination in some tissues of mice carrying the TRE-Cre allele. In fact, we found that Cre “leak” was independent of the reverse transactivator or doxycycline treatment, but instead was solely linked to the TRE-Cre allele. Ectopic Cre recombination was consistently observed to be most severe in tissues of the central nervous system and a similar degree of leak was observed in the progeny of multiple different TRE-Cre breeders. Ablating cells with aberrant TRE-driven Cre recombination resulted in stunted mice that were prone to developmental defects and exhibited significantly diminished survival. Researchers should take caution to consider potential side effects from ectopic recombination of their floxed alleles in nervous tissues. These findings highlight the need for proper controls in Cre systems and further development of the Tet-on/off systems in mice.

## 2 Methods

### 2.1 Animals

Mice expressing a reverse tetracycline transactivator driven by the adiponectin promoter (Adipo-rtTA), also known as AdipoChaser [10,16,17], were crossed to tetO-Cre mice (TRE-Cre, Jackson Labs #006234), which use a tetracycline response element (TRE) promoter to drive Cre expression in the presence of doxycycline (Figure 1A) [9].These Adipo-rtTA x TRE-Cre animals were then crossed to mTmG reporter mice, which express a floxed membrane-bound tdTomato red fluorescent protein followed by membrane-bound GFP (Jackson Labs #007676) [18]. Cre expression was induced in Adipo-rtTA/TRE-Cre/mTmG mice by placing them on a 600 mg/kg doxycycline-containing diet for 14 days (Bioserv S1407) [10,19]. Adipo-CreERT2 (Jackson Labs #025124) mice were crossed to mTmG (Jackson Labs #007676) to generate Adipo-CreERT2/mTmG mice, Cre expression was induced in these mice via five 50 mg/kg intraperitoneal injections of sterile tamoxifen in corn oil followed by a 10-day washout period [20]. To generate TRE-Cre/DTA mice, aforementioned male TRE-Cre mice (Jackson Labs #006234) were crossed to ROSA-DTA mice (Jackson Labs #009669) expressing diphtheria toxin fragment A downstream of a floxed STOP cassette [21]. All mice are from a C57BL/6 background; TRE-Cre, mTmG, and ROSA-DTA mice are C57BL/6J, while Adipo-CreERT2 are on a C57BL/6N background. Mice were euthanized by inhaled isoflurane overdose and death was assured via cervical dislocation and/or bilateral pneumothorax. All animal studies were approved by and conducted in compliance with policies of the University of Michigan Institutional Animal Care and Use Committee. Daily care of mice was overseen by the Unit for Laboratory Animal Medicine at the University of Michigan.

### 2.2 Whole mount confocal microscopy

Tissues were collected from mice and placed in light-protected tubes with ice-cold PBS then stored briefly on ice before imaging. Adipose tissues collected included interscapular brown (BAT), posterior subcutaneous white (psWAT), perirenal white (prWAT), gonadal white (gWAT), and bone marrow (BMAT) depots. Whole brains were dissected from the mice, then small pieces of the hypothalamus, prefrontal cortex, and cerebellum were further dissected for visualization. Samples were imaged in 1x PBS with a Nikon A1 confocal microscope and 20x objective.

### 2.3 Adipose tissue clearing and microscopy

**Figure 2:**
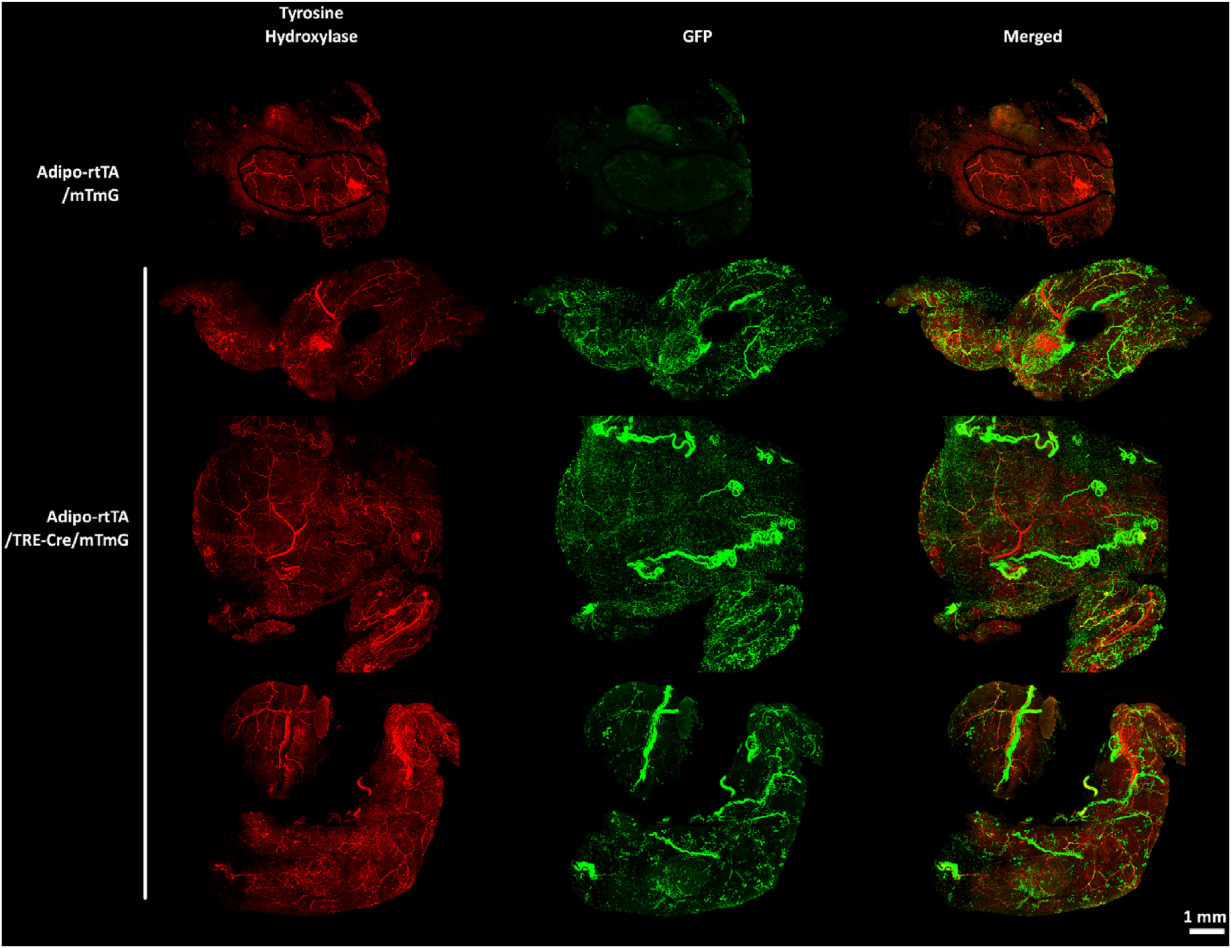
**Whole cleared psWAT depots reveal widespread rtTA-independent TRE-Cre activity in non-sympathetic nerve bundles.** Maximal intensity projections of cleared posterior subcutaneous white adipose tissue (psWAT) depots immunolabeled with antibody to tyrosine hydroxylase (red) and GFP (green) from male Adipo-rtTA/TRE-Cre/mTmG (N = 3) and Adipo-rtTA/mTmG (N = 1) mice. Scale bar = 1 mm, age 5–11 wks.

Whole psWAT depots from Adipo-rtTA/TRE-Cre/mTmG mice and TRE-Cre-negative controls were cleared and imaged according to the AdipoClear procedure developed by Chi et al. [22]. In brief, mice were transcardially perfused with PBS then 4% formaldehyde in PBS, followed by dissection of the psWAT depots. Tissues were dehydrated in a graded series of methanol washes, then delipidated with dichloromethane. Following rehydration in a reverse graded series of methanol washes, tissues were immunolabeled with primary antibodies against tyrosine hydroxylase (Abcam ab-112) and GFP (Abcam ab-13970). Tissues were then labeled with fluorophore-conjugated secondary antibodies excited at 568 and 647 nm, respectively. These fluorophore spectra were chosen to avoid strong collagen autofluorescence that is stimulated near 488 nm wavelengths. Tissues were embedded in 1% agarose and again dehydrated in a graded series of methanol washes, then cleared via additional incubations with dichloromethane. Finally, tissues were incubated with dibenzyl ether for refractive index matching. Embedded tissues were placed on #1.5 coverslips and imaged with a Nikon A1 point scanning confocal inverted microscope and 10x objective. Due to the slow scanning speed of point scanning confocal, line resolution for individual frames was set to 128 and pixel dwell to 4 Hz. 40 optical sections spaced 15 μm apart were collected, giving a total depth of 600 μm. Z-stacks of each field were stitched together to generate large volume 3-dimensional micrographs of whole psWAT depots. Figure 2 shows maximal intensity projections with the 568 nm channel detecting TH pseudocolored red and the 647 nm channel detecting GFP pseudocolored green.

### 2.4 Histology

Following dissection, soft tissues were fixed for 72 h in 10% neutral-buffered formalin at 4 °C then dehydrated in a graded series of ethanol washes. Bones were fixed for 24 h in 10% neutral-buffered formalin, then decalcified with 14% ethylenediaminetetraacetic acid (EDTA). Bones were then post-fixed in 14% EDTA, 4% formaldehyde for 24 h before dehydration in a graded series of ethanol washes. Tissues were processed and paraffin-embedded through the University of Michigan Orthopaedic Research Laboratory Histology Core facility, then sectioned at 5 μm thickness. Brains were sectioned along the midsagittal plane so cerebrum, cerebellum, and hypothalamus could be visualized in single sections. Sections were hematoxylin and eosin stained as previously described then imaged with an Olympus inverted microscope and 10x objective [23].

### 2.5 Statistics

All data are presented as mean ± S.D. Significance was determined using two-tailed Student's t-test when comparing two groups. An analysis of variance (ANOVA) was followed by post hoc analyses with Dunnett's or Sidak's test, as appropriate, when comparing multiple experimental groups. Observed differences were considered significant at p < 0.05 and are indicated with asterisks.

## 3 Results

### 3.1 Adipocyte-conditional Tet-on Cre recombination is similar in efficiency to tamoxifen-inducible model but with some ectopic recombination

Tamoxifen-inducible Cre models have proven themselves valuable tools that contribute spatiotemporal control to conditional knockout systems. However, tamoxifen itself has shown some undesirable lipotoxic and endocrine disrupting effects both *in vitro* and *in vivo* [7,8,[24], [25], [26], [27], [28]]. To avoid these potential effects, adiponectin-driven reverse tetracycline transactivator paired with a tetracycline response element-driven Cre (Adipo-rtTA/TRE-Cre or AdipoChaser) transgenic mouse model has been used as an alternative [10,16,17]. To confirm that these transgenic models reliably and specifically induce Cre recombination in adipocytes, both tamoxifen- and doxycycline-inducible models were crossed to mTmG dual fluorescent reporter mice, which express membrane-targeted tdTomato (mTomato) under basal conditions and EGFP (mGFP) following Cre recombination [18] (Figure 1A). Adipo-CreERT2 was activated with intraperitoneal tamoxifen whereas Adipo-rtTA/TRE-Cre was activated with doxycycline-containing chow. Whole mount confocal microscopy of freshly dissected adipose depots revealed a similar degree of EGFP expression in adipocytes between Adipo-CreERT2/mTmG mice and Adipo-rtTA/TRE-Cre/mTmG following Cre induction with tamoxifen or doxycycline, respectively (Figure 1B, Supplemental Fig. 1). While inspecting the tibial bone marrow compartment to determine the degree of Cre recombination in bone marrow adipocytes (BMAds), we noticed occasional EGFP-positive fibers resembling nerves coursing through the marrow of Adipo-rtTA/TRE-Cre/mTmG mice (Figure 1B, white arrowheads). Due to this finding, we searched for nonspecific Cre recombination within neuronal tissues. We observed varying degrees of ectopic Cre recombination, indicated by EGFP-positive cells, sporadically throughout brains of Adipo-rtTA/TRE-Cre/mTmG but not Adipo-Cre/mTmG mice (Figure 1B). These findings prompted us to characterize the nature of ectopic Cre recombination in Adipo-rtTA/TRE-Cre mice and to determine its cause, extent, and physiological implications in conditional deletion models.

### 3.2 Adipocyte-conditional Tet-on ectopic recombination occurs independently of doxycycline administration

To reveal the extent of ectopic recombination, including within adipose tissues, we searched for signs of leak in Adipo-rtTA/TRE-Cre/mTmG mice that were fed a normal chow diet with no doxycycline. Since Cre expression theoretically requires doxycycline-bound rtTA to activate TRE and drive Cre expression, there should be no mGFP in these mice. However, an extensive screen of tissues in these mice revealed sporadic mGFP expression in adipose, bone marrow, and muscle, and widespread Cre leak in the brain, spleen, and adrenal gland (Supplemental Figs. 2 and 3). We also noticed a higher degree of Cre recombination in adipocytes of the perirenal depot compared to gonadal, posterior subcutaneous, or brown adipose depots.

Unexpectedly, TRE-Cre/mTmG control mice lacking the doxycycline-inducible transactivator and fed normal chow diet also showed sporadic ectopic Cre recombination in white and brown adipocytes, and similar pattern of EGFP-positive fibers across multiple tissue types, including adipose, brain, and bone marrow (data not shown). Although limited to two mice, these findings suggested ectopic recombination occurs independent of doxycycline treatment or the adiponectin-rtTA transgene and led us to further investigate the TRE-Cre transgene.

White adipose tissues, particularly subcutaneous depots, are highly innervated by sympathetic inputs, which stimulate adipocyte lipolysis in response to a wide array of external cues, including fasting, cold exposure, glucoprivation, and exercise [[29], [30], [31], [32]]. Due to the importance of sympathetic innervation in posterior subcutaneous WAT (psWAT), we evaluated CRE recombination in chow-fed Adipo-rtTA/TRE-Cre/mTmG mice with immunofluorescent confocal microscopy of whole cleared psWAT depots against GFP and tyrosine hydroxylase (TH), the rate limiting enzyme of catecholamine synthesis and marker for sympathetic neurons. In agreement with our earlier observation, ectopic Cre recombination was noted in both psWAT adipocytes and nerve fibers while tissue lacking TRE-Cre had no specific GFP signal (Figure 2). However, we found very little overlap of GFP and TH signals suggesting TRE-Cre recombination does not specifically target sympathetic neurons. Yet, the functional importance of the cell population targeted by uncontrolled Cre may be consequential.

### 3.3 TRE-Cre causes ectopic recombination independent of reverse tetracycline transactivator (rtTA)

**Figure 3:**
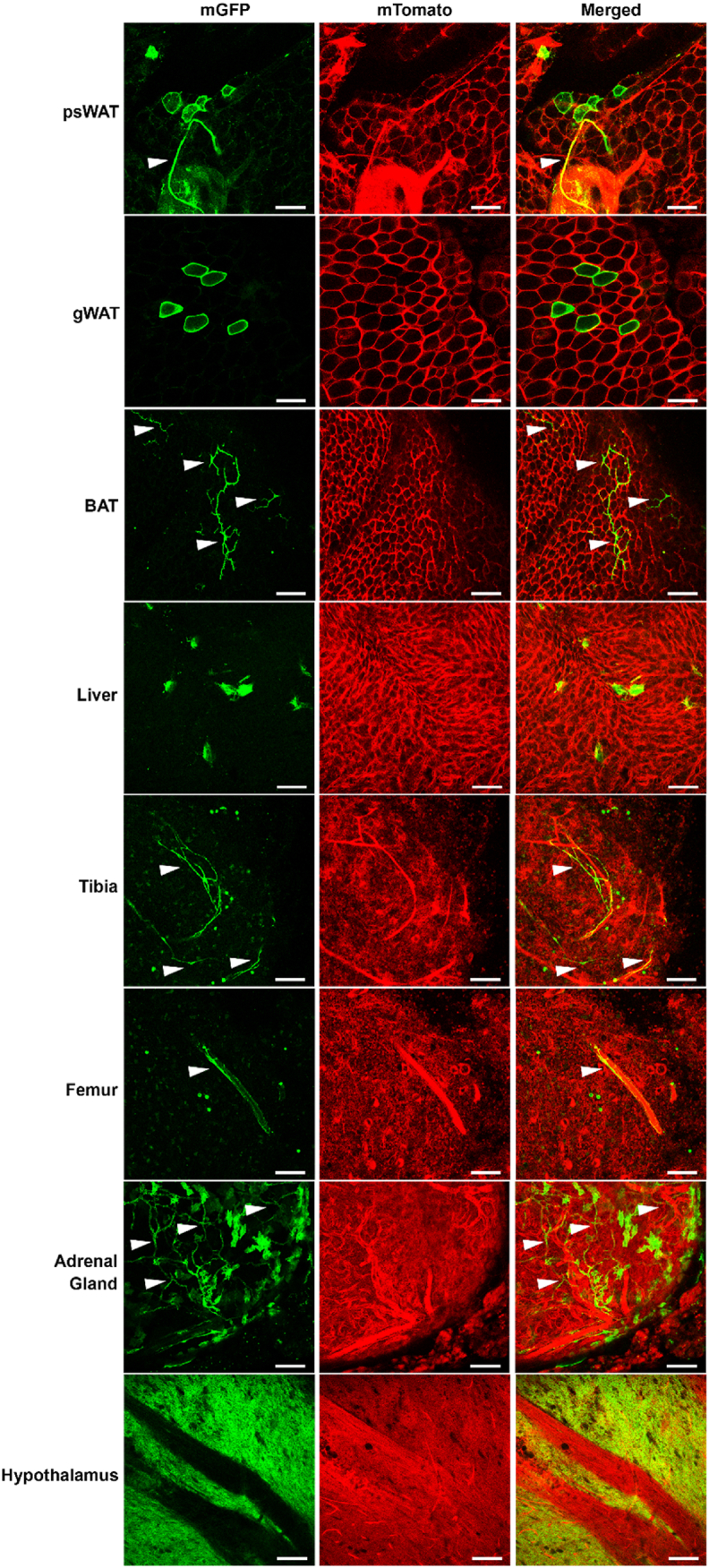
**TRE-Cre drives ectopic recombination in multiple tissues independent of reverse tetracycline transactivator (rtTA).** Confocal micrographs of freshly dissected mixed sex TRE-Cre/mTmG tissues (N = 5). mGFP is represented in green, mTomato is represented in red, and white arrowheads point to nerve fibers. Fields selected show maximal extent of nonspecific Cre recombination. Scale bar = 100 μm, age 13–15 wks.

The pTet promoter can be activated by residual rtTA in the absence of doxycycline in some cases [33,34], and nonspecific Cre recombination can be worsened by poor breeding strategies [35]; thus, we bred a cohort of TRE-Cre/mTmG mice using naïve male TRE-Cre breeders ordered directly from Jackson Laboratories. Wholemount confocal microscopy revealed sporadic ectopic Cre recombination in freshly dissected adipose tissues, liver, and bone marrow, where the maximal observable leak was contained to only a few cells per field (Figure 3, Supplemental Fig. 4). Consistent with our previous experiments, cells with Cre leak often appear to be nerve fibers (white arrowheads). However, neuroendocrine and neuronal tissues of the adrenal gland and hypothalamus, respectively, also exhibited widespread basal Cre recombination. The pattern of ectopic Cre recombination is nearly identical to that observed in Adipo-rtTA/TRE-Cre/mTmG mice (Supplemental Fig. 2), leading us to conclude that this leak is solely a function of TRE-Cre rather than the doxycycline-inducible transactivator.

### 3.4 TRE-Cre causes recombination in cells required for normal development of mice

**Figure 4:**
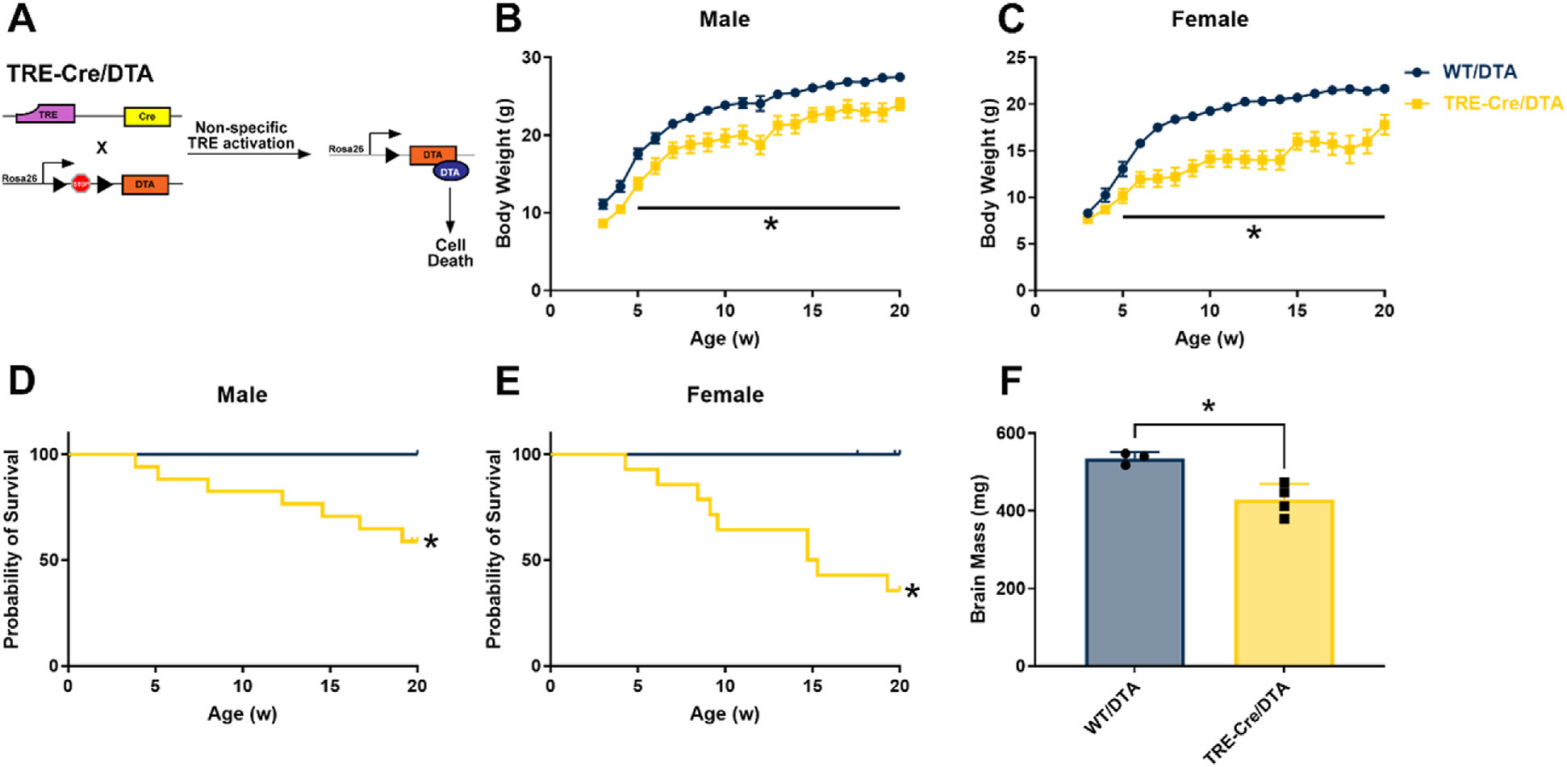
**TRE-Cre recombination occurs in cells required for normal development of mice.** Diagrammatic representation of TRE-Cre/DTA mice with genes represented as rectangles and proteins as ellipses (A). Body weight of male (B) and female (C) WT/DTA vs TRE-Cre/DTA mice from age 3–20 weeks. Survival curve of WT/DTA vs Tre-Cre/DTA male (D) and female (E) mice from 3 to 20 weeks of age. Brain mass of a subset of 20-week-old male WT/DTA vs Tre-Cre/DTA mice (F). ∗p < 0.05.

To understand the functional consequence of ectopic Cre recombination inherent to the TRE-Cre mouse, we crossed hemizygous TRE-Cre males to stop-floxed DTA female mice, which express cytotoxic diphtheria toxin fragment-A to ablate Cre expressing cells [21] (Figure 4A). Pups were born with normal appearance, no apparent sex differences, and inherited the TRE-Cre transgene at normal Mendelian ratios (Supplemental Figs. 5A and B). Although no differences were noted between the groups at weaning, by 5 weeks age TRE-Cre/DTA male and female mice were stunted relative to WT/DTA controls, as demonstrated by lower body weight (Figure 4B,C). We also observed multiple cases of hydrocephalus, malocclusion, and penile prolapse in TRE-Cre/DTA mice whilst the WT/DTA mice were unaffected. Perhaps due to the high incidence of developmental defects, both male and female TRE-Cre/DTA mice exhibited significantly reduced survival (Figure 4 D, E). Just 59% of male and 36% of female survived to 20 weeks of age, yet there were no deaths in the WT/DTA control group.

To investigate possible cause of death, a cohort of male TRE-Cre/DTA mice that survived to 20 weeks of age was dissected to probe gross or microscopic histological differences between the groups. Body weight did not differ between groups (Supplemental Fig. 5C). Mass of adipose depots (psWAT, gWAT, and BAT), liver, spleen, and adrenal gland were similarly unaffected, despite the high degree of ectopic Cre recombination previously noted in spleen and adrenal glands (Supplemental Figs. 5D–I). However, brain mass was significantly reduced in the TRE-Cre/DTA group (Figure 4F). This observation, along with noted cases of hydrocephalus, led us to speculate that ectopic Cre recombination in the brain is the main cause of dysfunction in these mice. Histological analysis of dissected tissues failed to reveal obvious differences between groups (Supplemental Fig. 6). Together, these data demonstrate that basal Cre recombination inherent to TRE-Cre mice targets a substantial population of cells within the central nervous system. If perturbed, loss of function of this cell population is sufficient to prevent normal development and/or viability of mice.

## 4 Discussion

In our work with TRE-driven Cre, we first noticed nonspecific Cre recombination in fibers resembling peripheral nerves, coursing through bone marrow and adipose tissue depots. The nervous system maintains bidirectional communication with adipose depots via sympathetic efferent and sensory afferent fibers [29]. Our observed lack of colocalization between markers for Cre recombination and sympathetic nerves (Figure 2), paired with only weak and controversial evidence of parasympathetic inputs in adipose [36], leads us to speculate that the observed nonspecific Cre recombination targets sensory nerves. In WAT, sensory nerves have been shown to regulate lipolysis, adipogenesis, and thermogenesis [36]. Unintentional perturbation of these processes via ectopic Cre recombination could obscure the true effect in adipocyte-specific conditional systems, thus fueling our desire to further characterize the nature of this ectopic recombination.

While ectopic Cre recombination in peripheral sensory nerves could certainly cause undesirable experimental effects, the extensive leak demonstrated in the central nervous system is more likely to cause deleterious effects in mice. Recent work by Jouvet et al. found nonspecific recombination of Cre in the brains of TRE-Cre mice and observed that leak was more widespread in female mice than males [37]. While we did not see a similar sex difference in our micrographs, survival of TRE-Cre/DTA female mice trended lower than in males (Figure 4D,E). However, our studies were not powered to detect sex specific differences. Future studies further probing sex specific differences of ectopic TRE-Cre recombination will provide valuable information for the field. Additionally, Jouvet et al. found extensive TRE-Cre leak in the gastrointestinal tract, including stomach duodenum, jejunum, ileum, and colon. In line with this observation, we observed that affected TRE-Cre/DTA mice lost considerable body weight prior to death, although this may also be secondary to cell death in the central nervous system, rather than impaired nutrient absorption. These considerations highlight the potential for leaky TRE-Cre to cause a phenotype when crossed with a floxed allele of interest. However, it should be noted that both mTmG and stop-floxed DTA transgenes used in our studies are inserted at the Rosa26 locus and are thus highly likely to have chromatin accessible to Cre recombinase; less accessible floxed alleles may have less severe ectopic recombination.

In light of potential confounding variables from ectopic TRE-Cre recombination and unpredictable degrees of ectopic recombination between different floxed alleles, proper experimental controls in these models are imperative. To ensure rigor and reproducibility between experiments, we recommend a two-step approach to control for ectopic TRE-Cre recombination. First, when establishing a new floxed allele of interest, researchers should validate that ectopic recombination is low or nonexistent under their intended experimental conditions. Tissues of TRE-Cre^−/−^/rtTA^+/^^−^/allele^fl/fl^/(+)Dox and TRE-Cre^+/^^−^/rtTA^−/^^−^/allele^fl/fl^/(+)Dox mice should be compared to TRE-Cre^−/−^/rtTA^−/^^−^/allele^fl/fl^/(+)Dox controls to screen for signs of recombination at the mRNA or protein levels (Graphical Abstract). Floxed alleles with defined roles in neuronal functioning should be screened with particular care, as neurons were the most common target for ectopic recombination in our studies. This validation step also provides an opportunity to screen for off-target effects of tTA or rtTA to rule out deleterious effects that have been noted in brain, lungs, β-cells, and T-cells [[37], [38], [39], [40]]. Second, experiments testing spatiotemporal excision of a validated floxed allele should use TRE-Cre^+/^^−^/rtTA^+/^^−^/allele^fl/fl^/(+)Dox mice for the experimental group and TRE-Cre^+/^^−^/rtTA^−/^^−^/allele^fl/fl^/(+)Dox for control. This design prevents both ectopic TRE-Cre recombination and doxycycline-induced dysbiosis from becoming independent variables between control and experimental groups.

An important goal moving forward with doxycycline inducible Cre models is to reduce nonspecific Cre recombination while retaining efficiency in the system. As with any Cre mouse model, employing proper breeding strategies is imperative for best results [41]. The Cre transgene must never be bred to homozygosity and careful attention should be paid toward possible parent-of-origin effects, as these variables can worsen ectopic recombination. One suggested approach to limit ectopic recombination is to screen strain founders for those with low rates of nonspecific recombination [42]. In our experiments we found similar patterns of TRE-Cre leak across progeny from five different male breeders, including young TRE-Cre mice (#006234) ordered directly from Jackson Laboratories. These findings suggest that ectopic Cre recombination in nervous tissues may be inherent to TRE-Cre mice, likely due to previous noted promiscuity of the pTet promoter which drives TRE-Cre [[43], [44], [45]]. However, since we only tested one TRE-Cre strain, ectopic recombination may also be related to the transgene insertion site.

Ectopic Cre recombination driven by nonspecific activation of pTet can occur via multiple unique mechanisms. In some cases, background activity at the pTet promoter can be triggered by rtTA transactivator binding in the absence of doxycycline [46], although multiple modified transactivators have been developed to limit this background activity while maintaining strong doxycycline sensitivity [33]. In the case of our TRE-Cre mouse strain of interest, we demonstrated a similar pattern of ectopic Cre recombination in the presence or absence of the Adipo-rtTA transactivator or doxycycline administration, suggesting background activity is solely a function of the pTet promoter (Figure 3, Supplemental Fig. 2). One approach to limit pTet background activation in mice is to incorporate a tetracycline-controlled transcriptional silencer (tTS) which silences the pTet promoter in the absence of tetracycline [42,47,48]. Tetracycline treatment (e.g., doxycycline) in pTet/rtTA/tTS systems releases the repression of pTet by tTS while simultaneously inducing its activation through rtTA.

Since the development of the TRE-Cre mouse used in this study, significant efforts have been made in minimizing pTet background expression. Second generation pTet promoters resulted from multiple modifications that were made to the original pTet (pTet-1) to minimize background whilst retaining strong inducibility. These modifications ultimately resulted in pTet-14, more commonly known as pTight, which features a truncated minimal CMV and fewer nucleotides between tetO sequences to limit positional effects of mammalian cis elements on the promoter [33,49]. In mice, pTight-driven LacZ reporter expression is undetectable in brains of advanced tTA Tet-off mice, demonstrating pTight has no intrinsic leak in the brain [50]. pTet has been further developed into the T-series, or third generation, Tet-responsive promoters. T-series pTet promoters retain the tetO spacing of the second-generation pTet-14 but randomized spacer sequences to minimize operator region symmetry, as palindromic sequences are not well suited for use with viral vectors [49]. Subsequent mutations of the minimal CMV moiety were made and these mutants were tested for background and induced activity. The sixth promoter mutant in this series, pTet-T6, proved the most effective, vastly outperforming both pTet-1 and pTet-14 in fold change induction between background and induced states [49]. While second and third generation pTet promoters are a promising solution to basal pTet activity observed in first generation TRE-Cre mice, it appears only pTight has been implemented in a mouse model but is not yet commercially available [50].

### 4.1 Conclusions

Herein we demonstrate ectopic Cre expression is inherent to TRE-Cre (#006234) transgenic mice and is particularly pronounced within the central nervous system. TRE-driven Cre expression occurs independently of doxycycline administration or transactivator presence. Ablation of cells expressing ectopic Cre is sufficient to impair the development and survival of mice. We suggest that future studies with these mice utilize a two-step validation and control approach, whereby floxed alleles are first screened for ectopic Cre recombination severity and off-target transactivator effects, then later experiments are controlled with TRE-Cre^+/^^−^/rtTA^−/^^−^/allele^fl/fl^/(+)Dox mice. We encourage the development of new TRE-Cre mouse lines which incorporate second or third generation pTet promoters to minimize basal Cre expression while maintaining high sensitivity and specificity.

## Author contributions

**K.T.L.:** Conceptualization, Methodology, Validation, Formal analysis, Investigation, Writing – Original Draft, Writing – Review & Editing, Visualization, Supervision. **L.R.O.:** Formal Analysis, Investigation, Writing -Review & Editing. **O.A.M.:** Conceptualization, Writing – Review & Editing, Supervision, Funding acquisition.

## Data availability

The authors confirm that the data supporting the findings of this study are available within the article and its associated supplemental material. Data not displayed within the article are available from the corresponding author, O.A.M., upon request.

## Funding information

Research for this study was supported by grants from the NIH to O.A.M. (R01 DK121759 and R01 DK125513) and K.T.L. (T32 DK071212 and F32 DK122654).
