# Supplementary material for: Tetracycline response element driven Cre causes ectopic recombinase activity independent of transactivator element
Source: Mol Metab. 2022 Apr 19;61:101501. doi: 10.1016/j.molmet.2022.101501 (PMC9170755; doi:10.1016/j.molmet.2022.101501)
Supplement: Multimedia component 1 [file mmc1.docx]

**Supplementary Materials: Tetracycline response element driven Cre causes ectopic recombinase activity independent of**

**transactivator element**

Kenneth T. Lewis^1^, Lily R. Oles^1^, and Ormond A. MacDougald^1,2^*

^1^University of Michigan Medical School, Department of Molecular & Integrative Physiology, Ann Arbor, MI

^2^University of Michigan Medical School, Department of Internal Medicine, Ann Arbor, MI

*Correspondence to: Ormond A. MacDougald

NCRC Building 25, Rm. 3686

2800 Plymouth Rd., Ann Arbor, MI 48105

Tel:(734) 647-4880

E-mail: macdouga@umich.edu


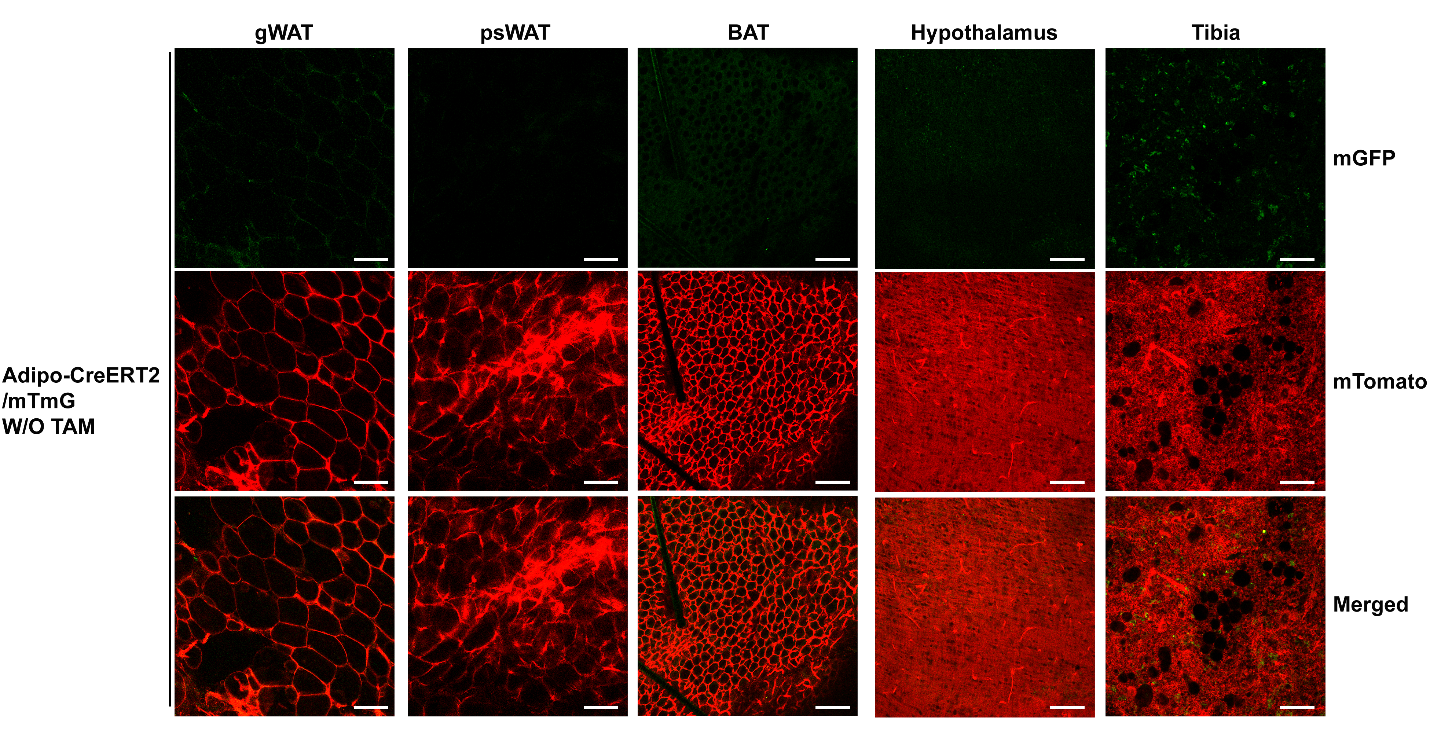


**Supplemental Figure 1: Contol for Figure 1.** Confocal micrographs of freshly dissected female Adipo-CreERT2/mTmG tissues (N=1) from a mouse that had not been administered tamoxifen. Autofluorescence emitting near 488 nm is represented in green and mTomato is represented in red. Scale bar = 100 µm, age 29 wks.


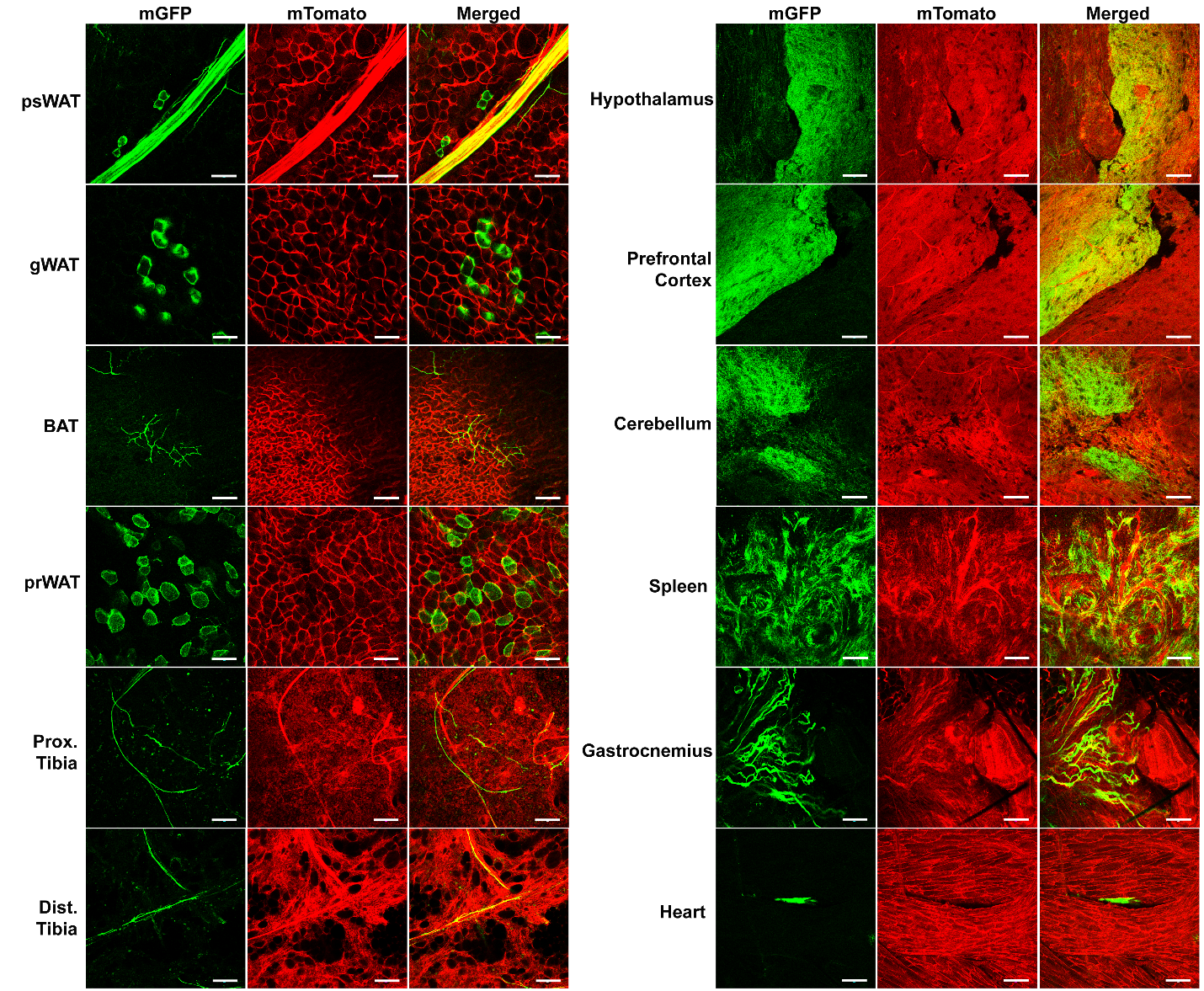


**Supplemental Figure 2: TRE-Cre drives ectopic recombination in multiple tissues independent of doxycycline.** Confocal micrographs of freshly dissected adipose tissues, brain (hypothalamus, prefrontal cortex, and cerebellum), spleen, skeletal muscle (gastrocnemius), and heart of chow-fed male Adipo-rtTA/TRE-Cre/mTmG tissues (N=4). Adipose tissues include posterior subcutaneous white (psWAT), gonadal white (gWAT), brown (BAT), perirenal white (prWAT), and bone marrow (proximal and distal tibia) depots. mGFP is represented in green and mTomato is represented in red. Fields selected show maximal extent of nonspecific Cre recombination. Scale bar = 100 µm, age 4-11 wks.


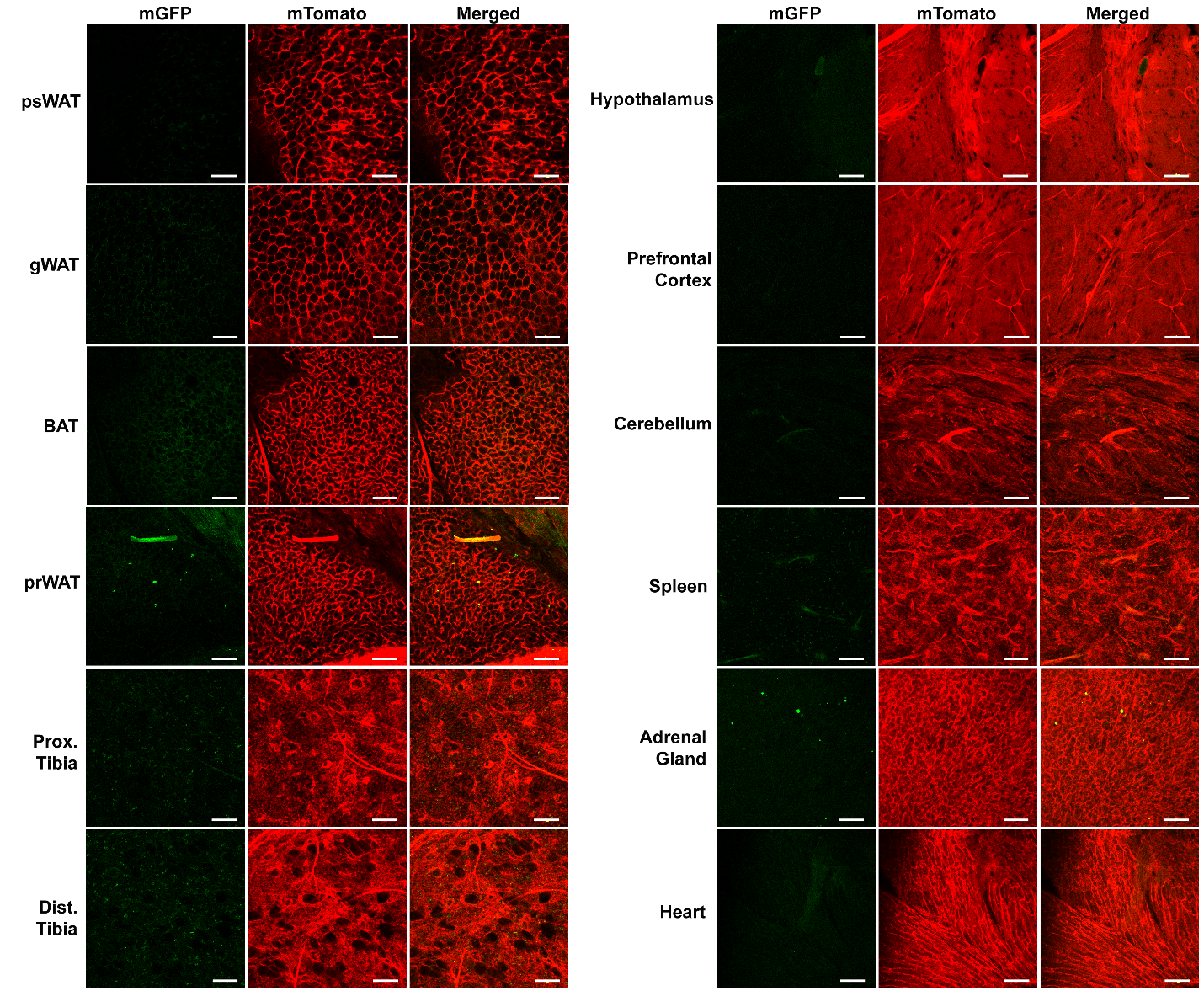


**Supplemental Figure 3: Control for Supplemental Figure 2.** Confocal micrographs of freshly dissected male Adipo-rtTA/mTmG tissues (N=2). Autofluorescence emitting near 488 nm is represented in green and mTomato is represented in red. Scale bar = 100 µm, age 5-12 wks.


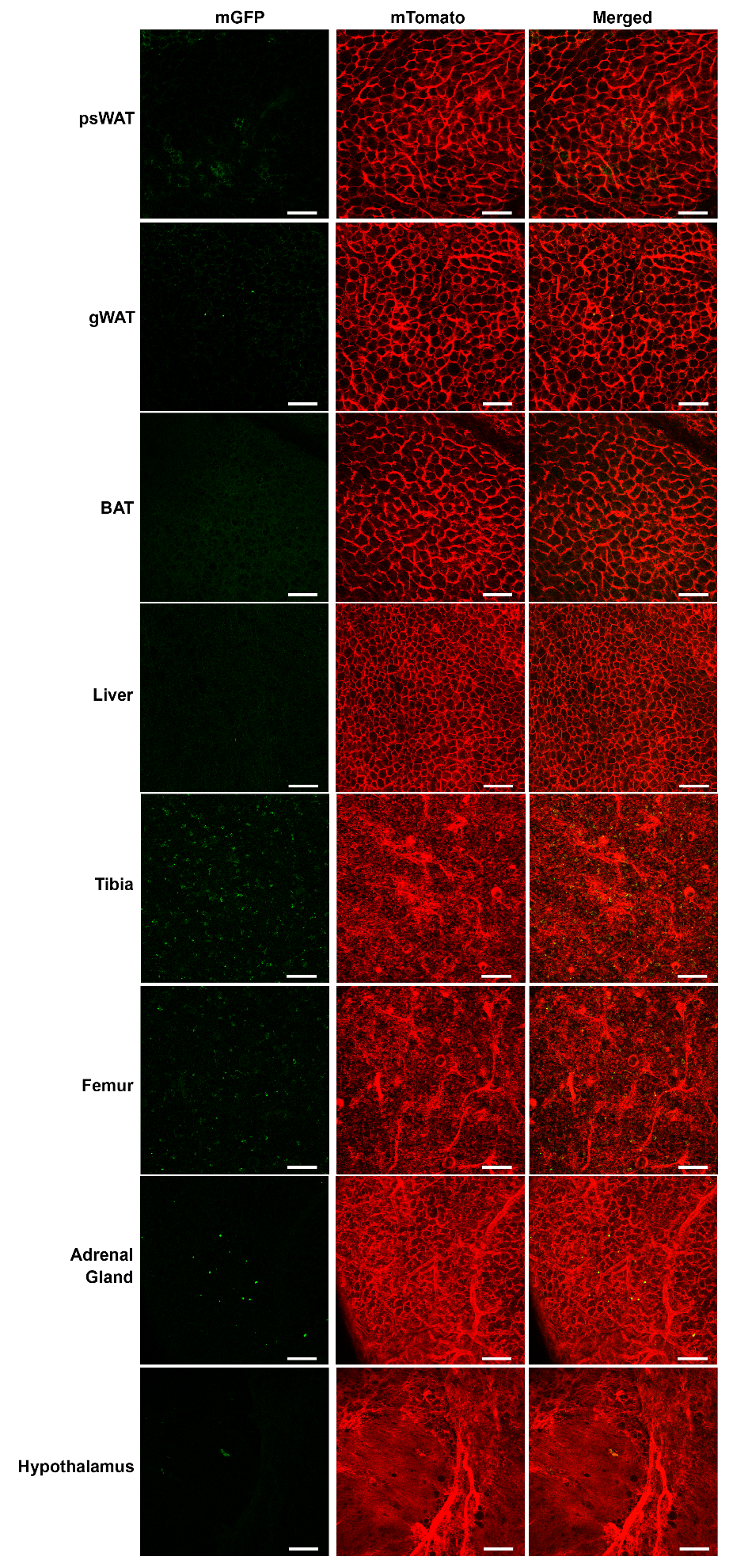


**Supplemental Figure 4: Control for Figure 3.** Confocal micrographs of freshly dissected female Adipo-rtTA/mTmG tissues (N=3). Autofluorescence emitting near 488 nm is represented in green and mTomato is represented in red. Scale bar = 100 µm, age 7-23 wks.

**
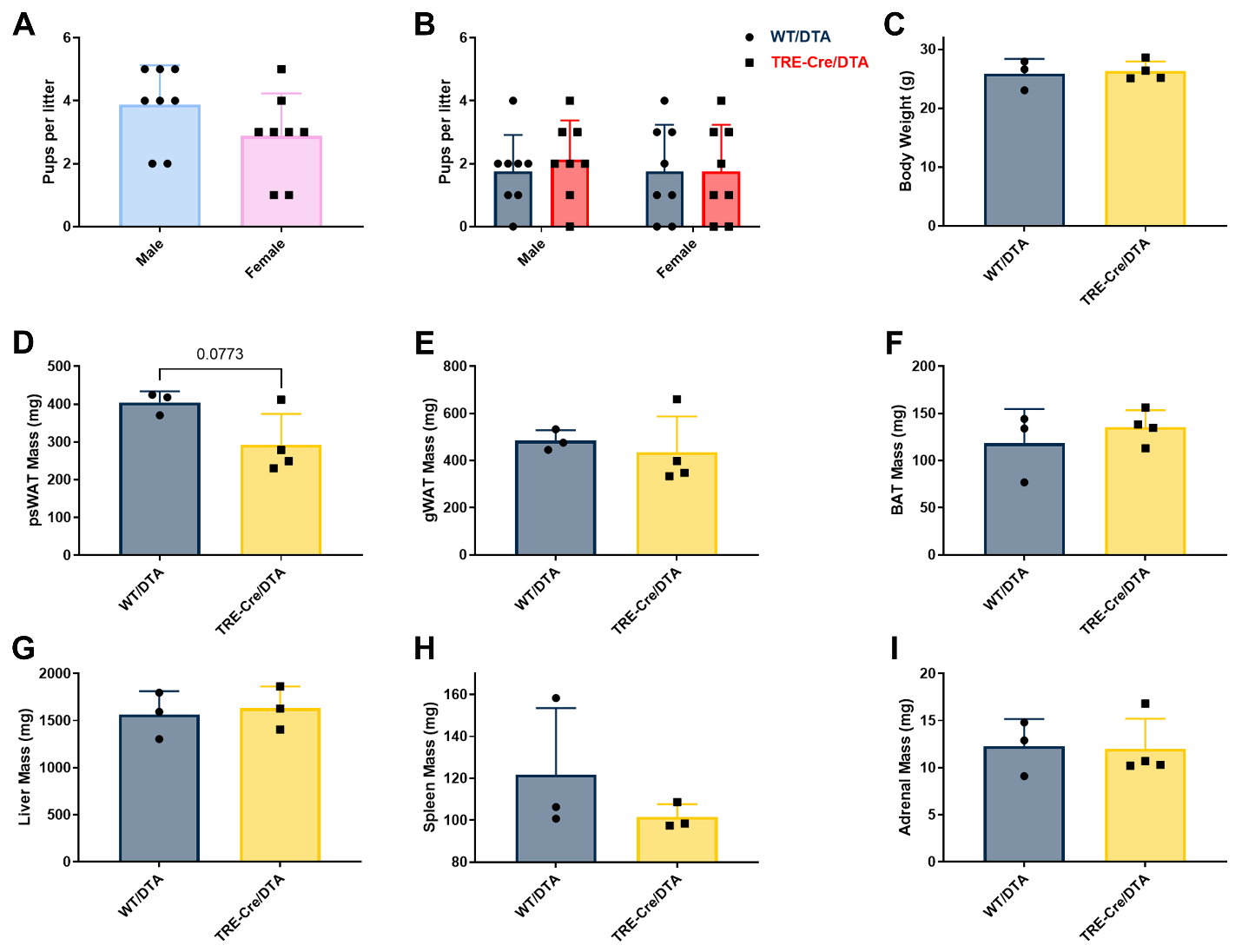
**

**Supplemental Figure 5: TRE-Cre/DTA birth records and adult tissue mass.** Sex distribution (A) and TRE-Cre transgene inheritance (B) in TRE-Cre/DTA litters. Body weight (C) and tissue mass for posterior subcutaneous white adipose (psWAT) (D), gonadal white adipose (gWAT) (E), (interscapular brown adipose (BAT) (F), liver (G), spleen (H), and adrenal glands (I) of a subset of male TRE-Cre/DTA (N=4) and control (N=3) mice at 20 weeks of age.


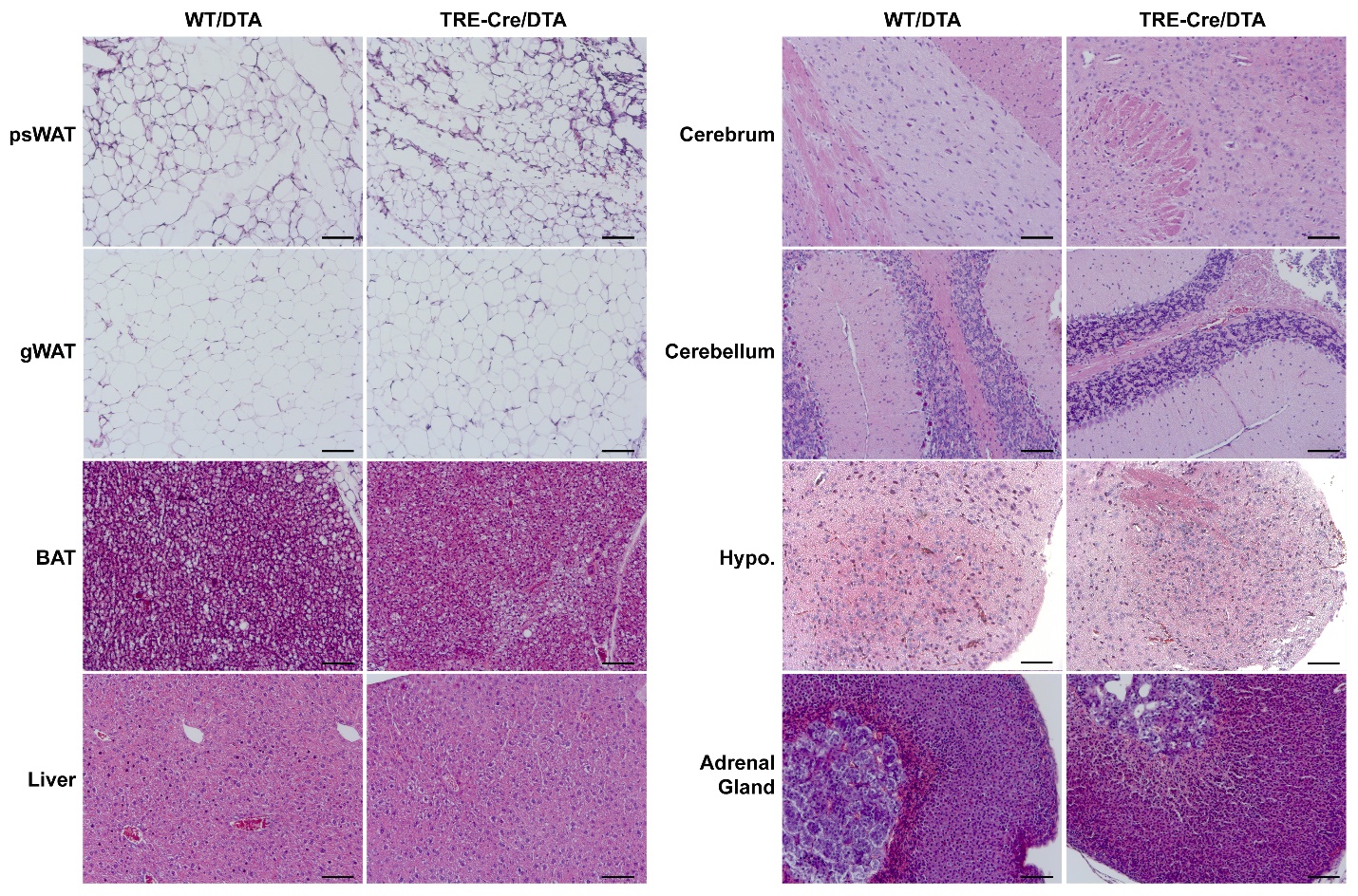


**Supplemental Figure 6: Adult TRE-Cre/DTA tissues appear histologically similar.** Representative brightfield micrographs of Hematoxylin & Eosin-stained paraffin-embedded tissue sections from male WT/DTA (N=3) and TRE-Cre/DTA (N=4) male mice, including midsagittal sections of cerebrum, cerebellum, and hypothalamus (Hypo.). Scale bar = 100 µm, age 20 wks.
